# Supplementary material for: Validity of gout diagnosis in Swedish primary and secondary care - a validation study
Source: BMC Musculoskelet Disord. 2015 Jun 16;16:149. doi: 10.1186/s12891-015-0614-2 (PMC4466844; doi:10.1186/s12891-015-0614-2)
Supplement: Additional file 1: Table S1. — Frequencies of patients who had one, two, or more main gout diagnoses and who met the cutoffs of the different classification criteria. [file 12891_2015_614_MOESM1_ESM.docx]

Validity of gout diagnosis in Swedish primary and secondary care – a validation study

Mats Dehlin*, Kalliopi Stasinopoulou, Lennart Jacobsson

Department of Rheumatology and Inflammation Research, Institute of Medicine, Sahlgrenska Academy at the University of Gothenburg, P.O. Box 480, 405 30 Gothenburg, Sweden

Email: mats.dehlin@vgregion.se; kalliopi.stasinopoulou@vgregion.se; lennart.jacobsson@gu.se

*Corresponding author: address as above, Tel: +46 732 520451, Email: mats.dehlin@vgregion.se

Abstract

Background

The diagnostic golden standard for gout is to detect monosodium urate (MSU) crystals in synovial fluid. While some gout classification criteria include this variable, most gout diagnoses are based on clinical features. This discrepancy between clinical practice and classification criteria can hinder gout epidemiological studies. Here, the objective was to validate gout diagnoses (International Classification of Diseases (ICD)-10 gout codes) in primary and secondary care relative to five classification criteria (Rome, New York, ARA, Mexico, and Netherlands). The frequency with which MSU crystal identification was used to establish gout diagnosis was also determined.

Methods

In total, 394 patients with ≥1 ICD-10 gout diagnosis between 2009 and 2013 were identified from the medical records of two primary care centers (n=262) and one secondary care center (n=132) in Gothenburg, Sweden. Medical records were assessed for all classification criteria.

Results

Primary care patients met criteria cutoffs more frequently when ≥2 gout diagnoses were made. Even then, few primary care patients met the Rome and New York cutoffs (19% and 8%, respectively). The ARA, Mexico, and Netherlands cutoffs were met more frequently by primary care patients with ≥2 gout diagnoses (54%, 81%,

and 80%, respectively). Mexico and Netherlands cutoffs were met more frequently by the rheumatology department patients (80% and 71%, respectively), even when patients with only 1 gout diagnosis were included. Analysis of MSU crystals served to establish gout diagnoses in only 27% of rheumatology department and 2% of primary care cases.

Conclusions

If a patient was deemed to have gout at ≥2 primary care center or ≥1 rheumatology-center visits according to an ICD-10 gout code, the positive predictive value of this variable in relation with the Mexico and Netherlands classification criteria was ≥80% for both primary care and rheumatology care settings in Sweden. MSU crystal identification was rarely used to establish gout diagnosis.

Keywords: gout, classification criteria, validity of diagnosis

Background

Gout is characterized by increased levels of uric acid in the blood, which lead to the accumulation of monosodium urate crystals (MSU) in the joints and tissue. This induces a strong inflammatory reaction that causes great pain. Gout may affect any joint, but it most commonly affects the lower extremities and has a nocturnal onset. The inflammation generally subsides within 2 weeks. Gout is the most common inflammatory arthritic disease; its worldwide prevalence is 1–2% [1-4], although prevalence rates vary greatly depending on genetic and cultural factors.

Since 1961, the golden standard for diagnosing gout has been the detection of intracellular (IC) MSU crystals by polarized light microscopy of the synovial fluid (SF) from the affected joint [5, 6]. However, studies have indicated that this method is rarely used for diagnosis in clinical practice [7, 8].

Gout has been diagnosed in the past by using several classification criteria (*Table 1*). These were developed on the basis of expert opinion to facilitate epidemiological studies and improve comparability between studies. The earliest were the 1963 Rome [9] and 1966 New York (NY)[10] criteria, which relied largely on the presence of tophi and the detection of IC MSU crystals in the SF (*Table 1*). Subsequent criteria, namely, the 1977 ARA [11] and the 2010 Mexico [12] criteria, incorporated several clinical characteristics of gout that are often used in clinical practice, such as monoarthritis with rapid onset and pronounced signs of inflammation. The Netherlands criteria were published in 2010, emphasizing clinical parameters and not primarily considering synovial fluid analysis [13]. Despite all the existing criteria, there is still a need for new classification criteria, which are currently being developed by ACR/EULAR (11). These new criteria are likely to be based on the

elements used by previous classification systems because several of these features were found to highly and accurately discriminate between patients with and without gout in a Delphi exercise conducted in 2013 involving both patients and physicians [14]. The new algorithm is also likely to include criteria based on new imaging techniques [15].

Several studies conducted over the past two decades have shown that the prevalence of gout is rising [16-18]. This highlights the importance of large-scale epidemiological studies that aim to identify the risk factors for developing gout and for poor gout outcomes, such as coronary vascular disease and death. Epidemiological studies that assess the health and economic consequences of gout are also warranted. Such studies could be based on medical record databases. However, before the International Classification of Diseases (ICD) codes for gout can serve as data sources for epidemiological studies, the validity of these codes must be determined. Several studies show that the validity of ICD codes for gout ranges from modest [19, 20], in American medical record databases, to fair, in UK databases [21, 22].

Sweden has a number of health care registers that serve as unique national sources of data for large epidemiological studies. In particular, the Swedish National Patient Register (NPR), which was established in 1964, contains complete national inpatient medical records since 1987. Furthermore, a recent study showed that by 2011, 99% of all annual somatic and psychiatric hospital discharges were registered in the inpatient part of the NPR [23]. In 2001, an outpatient register for secondary care was added to the NPR. By 2011, 87% of annual secondary care outpatient visits were recorded in the NPR [24]. Both registers have been used extensively for epidemiological research. The ICD codes for several diseases have a high

diagnostic validity, with positive predictive values (PPV) ranging between 85 and 95% [23]. For example, the PPVs for the diagnosis of myocardial infarction and rheumatoid arthritis were 98 [25] and 95% [26], respectively. However, the validity of ICD codes for diagnosing gout has not yet been assessed.

The primary objective of the present study was to evaluate the validity of ICD-10 codes for gout diagnosis in Sweden, as indicated by calculating the PPVs relative to the five classification criteria. The secondary objective was to determine the frequency with which the presence of IC MSU crystals in the SF was used to diagnose gout.

Methods

Setting

The study population consisted of all patients who were diagnosed with gout between 2009 and 2013 in two primary care centers and one specialized rheumatology department with in- and outpatient care in Gothenburg. Gothenburg is a city in western Sweden with approximately 533,000 inhabitants as of 2013. In Sweden, all inhabitants have a personal identification number that is used to register their health care visits in the NPR. The NPR for Gothenburg also includes primary care visits. The medical data that are collected in the NPR include primary and secondary diagnoses. However, the present study only considered the primary diagnoses. Since 1997, all diagnoses have been registered according to the Swedish version of the ICD-10.

The present study included the records of two primary care clinics (Olskroken and Masthugget) because the majority of patients with gout are usually diagnosed and treated by physicians in primary care centers. The two primary care clinics were chosen from the 30 primary care centers in Gothenburg. They both represent midsize primary care clinics in average income areas, with approximately 17000 (Olskroken) and 8000 (Masthugget) enlisted patients, respectively. Moreover, because more severe cases may be referred to specialized rheumatology clinics, the records from the only clinic in the area that provides specialized rheumatology care were also reviewed. This clinic is the largest rheumatology unit in the area with approximately 7500 patients enlisted and 15000 appointments per year. Ethical approval for the study was received from the Ethical Review Board of Gothenburg, Sweden. Informed consent from the patients was not needed since the data were studied in a group level and were anonymized.

Selection of cases and review of clinical records

Between 2009 and 2013 in Olskroken and Masthugget, 173 and 89 patients in total were diagnosed with gout at least once, respectively. During the same period, 132 patients were diagnosed with gout in the specialized rheumatology department at Sahlgrenska University Hospital. Only four patients included were present in both the patient selections from the primary care center and the rheumatology department.

Two rheumatologists reviewed the electronic medical records from the primary care centers and one trained research nurse assistant and two rheumatologists reviewed the records from the specialized rheumatology department. All reviews were performed according to a structured protocol that assessed all variables in the Rome, NY, ARA, Mexico, and Netherlands classification criteria, which are listed in

Table 1. Furthermore, information regarding age, sex, and comorbidity was extracted. Patients were considered to have comorbidity if it was mentioned in the clinical record or they were prescribed medication for a comorbidity.

The PPV was calculated by dividing the number of patients who met each set of the classification criteria (each of which are considered gold standards) by the number of patients who were diagnosed with gout in the medical record. Sensitivity and specificity were not calculated because this would have required a second population to determine the proportions of false and true negatives.

Statistical analyses

The primary care group (including the patients from both primary care centers) and the secondary care group were analyzed separately. Descriptive statistics were used to summarize the demographic and clinical characteristics of the two groups. When comparing categorical data, χ^2 test or, when appropriate, Fisher's exact test, were used. All statistical analyses were performed using IBM SPSS version 22 and SAS 9.3.

Results

Patient characteristics

In total, 394 patients were diagnosed at least once with an ICD-10 code indicating gout during the study period (2009–2013), and their medical records were reviewed. Of these, 262 were diagnosed at the two primary care centers, Olskroken and Masthugget; Olskroken had 173 patients (1.0% of the approximately 17000 patients enlisted to this center during the study period) and Masthugget had 89 patients (1.1% of the approximately 8000 patients enlisted to this center during the study period).

Of the 262 primary care group patients, 198 (76%) were men and 64 (24%) were women. On average, the men were younger (median 63.5, range 31–97 years) than the women (median 66.5, range 43–98 years). The total primary care group had a median age of 72.5 (range 31–98) years. The majority of the primary care group patients (n=155, 59%) had hypertension. Eighty-eight patients (34%) had cardiovascular disease, 61 (23%) had diabetes, and 160 (61%) were being or had been treated with allopurinol (*Table 2*).

In total, 132 patients were diagnosed at least once with an ICD-10 code indicating gout during the study period in the specialized rheumatology department at Sahlgrenska University Hospital. Of these, 106 (80%) were men and 26 (20%) were women. The men were younger (median 66.5, range 26–91 years) than the women (median 71, range 34–94 years). The total population had a median age of 71 (range 26–94) years. The majority (n=92, 70%) had kidney disease and/or hypertension (n=86, 59%). Fifty-four (41%) had cardiovascular disease, 25 (19%) had diabetes,

nine (7%) had undergone organ transplantation (liver or kidney), 14 (11%) had psoriasis, and 96 (73%) were being or had been treated with allopurinol (*Table 2*).

Validity of ICD-codes of gout

The patients with ≥1, ≥2, or ≥3 ICD-10 gout diagnoses in the medical records were then assessed for their ability to meet the cutoffs of each of the five classification criteria. Table 3 shows the PPVs for the two groups relative to each criteria set. The PPV patterns of the two groups were generally similar and increased with number of visits with a diagnosis of gout, although the patients in the rheumatology group were generally more likely to meet the criteria cutoffs than the primary care patients. In both groups, patients with a higher number of visits with a gout diagnosis had higher PPVs of meeting the criteria cutoffs for all criteria evaluated. Compared with those with ≥2 visits, those with only one visit with a main diagnosis of gout had significantly lower PPVs ($P \leq 0.03$) for all criteria evaluated (see Suppl. Table 1). The frequency of patients in the primary care group who met the Rome and NY cutoffs was very low. Only 19% and 8% of the patients with ≥2 gout diagnoses met these cutoffs, respectively. The frequency of primary care patients meeting the ARA cutoff was higher, especially in cases with ≥2 gout diagnoses (PPV=54%). The frequency of patients meeting the Mexico and Netherlands cutoffs was very high: approximately 80% of patients in both groups met the cutoffs for these criteria when ≥2 gout diagnoses were present.

Clearly, having ≥1 ICD-10 gout diagnosis did not yield sufficient validity in the primary care setting. For all criteria, the PPVs were less than 50%. In the rheumatology group, having ≥1 gout diagnosis yielded high PPVs, ranging from 61% (NY) to 80% (Mexico). Likewise, but to a lesser degree than in the primary care

setting, the PPVs were significantly ($P \leq 0.02$) lower in those with only one visit with a main diagnosis for gout compared to those with ≥2 gout diagnoses, except for the Netherlands criteria (see Suppl. Table 1).

Validity of individual features

As shown in Table 4, SF analysis was rarely used to diagnose gout in primary care. Only 6 (2%) of the 262 primary care patients underwent this test. The test was somewhat more common in the rheumatology department. Still, only 35 of the 135 patients (27%) underwent SF analysis to establish the diagnosis. Similarly, only 6 primary care patients (2%) and 31 rheumatology patients (23%) had a documented presence of tophi. However, most patients were assessed for serum levels of uric acid (78% of primary care patients and 95% of the rheumatology patients). The majority of patients tested for serum uric acid levels were positive (88% of tested primary care patients and 78% of tested rheumatology patients). Thus, in most cases, the diagnoses were based on clinical variables. The five most common clinical variables mentioned in the medical records were monoarthritis, first metatarsophalangeal (MTP) joint arthritis, redness over joints, two or more attacks of arthritis, and unilateral MTP 1 arthritis (*Table 5*).

Discussion

The present study showed that when an ICD-code for gout was recorded in at least two patient visits to a primary care center and at least one patient visit to a rheumatology department, the diagnosis had relatively high validity (a PPV of 80% or more) when compared to recent classification criteria like the Mexico and Netherlands criteria. The SF analysis was rarely used to establish a diagnosis in primary care. It was also only used in a minority of secondary care cases to establish the diagnosis.

Several studies have examined the validity of gout diagnosis relative to different classification criteria. When Malik *et al*. [19] examined the medical records of 289 patients in a Veteran's Health database, who had at least two ICD-9 coded episodes of gout, 36% met the ARA criteria. However, in the subgroup of 115 patients who were assessed by a rheumatologist, 83 (73%) met the ARA criteria. A similar study by Harrold *et al.* [20] was based on a random sample of the 800,000 patients in four managed care plans. The analysis of the chart reviews of 200 randomly selected patients who had two ICD-9 coded episodes of gout revealed that 121 were rated by physician consensus as having probable/definite gout. Thus, the PPV of $\geq$2 coded diagnoses of gout was 61%. However, there was low concordance between the physician assessments and the ARA, Rome, and NY criteria (κ = 0.17, 0.16, and 0.20, respectively).

A key criterion in the Rome and NY algorithms is the presence of tophi. Moreover, of the four NY algorithm criteria, one is the presence of MSU crystals in the SF. This

makes these two algorithms difficult to use in the primary care setting, which is where the vast majority of gout cases are diagnosed. Indeed, a large prospective epidemiological study of gout, the Health Professionals study, revealed that SF analysis was only performed in 11% of the participants who had a diagnosis of gout [3, 4]. However, unlike these earlier criteria sets, the Mexico and Netherlands criteria do not rely on SF analyses. This probably explains why ICD-10 gout diagnosis in the primary care setting had good PPVs in our study when compared to these latter algorithms.

When Roddy *et al*. (2010) [22] identified primary care consultations for acute gout in two primary care databases by free-text screening of the medical records, 583 patients were deemed to have consulted for acute gout. However, the medical records only mentioned features that were suggestive of acute gout in 312 (55%) of these patients. Hence, the quality of the medical records is crucial. Notably, the differences in our study between the primary and secondary care medical records regarding PPV have also been observed by other studies [27, 28]. Thus, the PPV of rheumatic diagnoses seems to be influenced by the medical specialty of the health care provider.

In the present study, the lack of documentation of the indications for urate-lowering therapy in the medical records in primary care presented a problem, particularly for patients with chronic stable gout without tophi and infrequent acute joint symptoms. In such cases, especially if patients lack symptoms and possibly have normal serum uric acid levels, the administration of allopurinol may support the diagnosis of gout.

This stresses the need for new classification criteria that accounts for intercritical or chronic gout.

The strengths of the present study include the fact that the medical records from both primary and secondary care settings were reviewed. This allowed us to compare the two settings in terms of gout diagnosis validity. Furthermore, our computerized population-based registers enabled us to retrieve and review all medical records from all registered patients with gout within the defined geographical area and time frame for both the primary and secondary care providers.

The limitations of the present study include the possibility that not all patients with gout in the given geographical area during the study period were diagnosed. It is also possible that some patients with gout were not cared for by public health care providers. However, since less than 13% of the population of Sweden is cared for in the private health sector [24], the latter patients are likely to have only a limited effect on the generalizability of our patient sample. The uncertainty of how representative our sample is of the general population with gout is another limitation of this study, one which we are presently addressing as part of a large epidemiological study of gout prevalence in western Sweden. Furthermore, there was a lack of relevant information because of insufficient recording or the lack of performing relevant tests. However, if this information had been available, it would have likely increased the rates of criteria fulfillment. Last but not least, the validity discussed in this paper is limited because only the PPVs were calculated. We were unable to calculate the sensitivity, specificity, negative predictive value, likelihood ratio, and reliability of the

ICD-10 gout codes. Further studies on the full validity of the ICD-10 gout codes are warranted.

Conclusions

In conclusion, this study showed that when patients were deemed to have gout after ≥2 gout diagnoses in primary care and ≥1 gout diagnoses in secondary care, these diagnoses had relatively high validity compared with the clinically based Mexico and Netherlands classification criteria. Moreover, the gout diagnoses in both settings were largely based on clinical parameters; analyses of MSU crystals in the SF or documentation of tophi were rarely performed in both primary and secondary care.

List of abbreviations

MSU = monosodium urate

SF = synovial fluid

NPR = National Patient Register

Competing interests

The authors declare that they have no competing interests.

Author contributions

MD helped to plan the study, collected the medical records, analyzed the data, and drafted the manuscript. KS helped to plan the study, collected the medical records, analyzed the data, and helped to draft the manuscript. LJ planned the study, collected the medical records, analyzed the data, and helped to draft the manuscript. All authors read and approved the manuscript.

Acknowledgements

We would like to thank the staff of the Masthugget and Olskroken primary care units for their support in obtaining the medical records, and research nurse assistant Cecilia Jacobsson for participating in reviewing the clinical records. This work has been funded by grants from Reumatikerfonden, the Felix Neubergh Foundation, the

Inger Bendix Foundation for Medical Research, the Hjalmar Svenssons Research Fund, and Gothenburg University.

References

1. Kuo CF, Grainge MJ, Mallen C, Zhang W, Doherty M. Rising burden of gout in the UK but continuing suboptimal management: a nationwide population study. Ann Rheum Dis. 2015;74(4):661-7. doi:10.1136/annrheumdis-2013-204463.

2. Zhu Y, Pandya BJ, Choi HK. Prevalence of gout and hyperuricemia in the US general population: the National Health and Nutrition Examination Survey 2007-2008. Arthritis Rheum. 2011;63(10):3136-41. doi:10.1002/art.30520.

3. Smith E, Hoy D, Cross M, Merriman TR, Vos T, Buchbinder R et al. The global burden of gout: estimates from the Global Burden of Disease 2010 study. Ann Rheum Dis. 2014;73(8):1470-6. doi:10.1136/annrheumdis-2013-204647.

4. Roddy E, Choi HK. Epidemiology of gout. Rheumatic diseases clinics of North America. 2014;40(2):155-75. doi:10.1016/j.rdc.2014.01.001.

5. McCarty DJ, Hollander JL. Identification of urate crystals in gouty synovial fluid. Ann Intern Med. 1961;54:452-60.

6. Pascual E, Batlle-Gualda E, Martinez A, Rosas J, Vela P. Synovial fluid analysis for diagnosis of intercritical gout. Ann Intern Med. 1999;131(10):756-9.

7. Choi HK, Atkinson K, Karlson EW, Willett W, Curhan G. Alcohol intake and risk of incident gout in men: a prospective study. Lancet. 2004;363(9417):1277-81. doi:10.1016/S0140-6736(04)16000-5.

8. Choi HK, Atkinson K, Karlson EW, Curhan G. Obesity, weight change, hypertension, diuretic use, and risk of gout in men: the health professionals follow-up study. Arch Intern Med. 2005;165(7):742-8. doi:10.1001/archinte.165.7.742.

9. Kellgren J H JM, Ball JF. The epidomiology of chronic rheumatism. Oxford: Blackwell Scientific. 1963.

10. Decker J. Report from the subcommittee on diagnostic criteria for gout. In: Bennett PH, Wood PHN, eds. Population studies of the rheumatic diseases. Proceedings of the Third International Symposium, New York, June 5-10, 1966. Amsterdam: Excerpta Medica Foundation,. 1968;1968:385-7.

11. Wallace SL, Robinson H, Masi AT, Decker JL, McCarty DJ, Yu TF. Preliminary criteria for the classification of the acute arthritis of primary gout. Arthritis Rheum. 1977;20(3):895-900.

12. Pelaez-Ballestas I, Hernandez Cuevas C, Burgos-Vargas R, Hernandez Roque L, Teran L, Espinoza J et al. Diagnosis of chronic gout: evaluating the american college of rheumatology proposal, European league against rheumatism recommendations, and clinical judgment. J Rheumatol. 2010;37(8):1743-8. doi:10.3899/jrheum.091385.

13. Janssens HJ, Fransen J, van de Lisdonk EH, van Riel PL, van Weel C, Janssen M. A diagnostic rule for acute gouty arthritis in primary care without joint fluid analysis. Arch Intern Med. 2010;170(13):1120-6. doi:10.1001/archinternmed.2010.196.

14. Prowse RL, Dalbeth N, Kavanaugh A, Adebajo AO, Gaffo AL, Terkeltaub R et al. A delphi exercise to identify characteristic features of gout - opinions from patients and physicians, the first stage in developing new classification criteria. J Rheumatol. 2013;40(4):498-505. doi:10.3899/jrheum.121037.

15. Dalbeth N, Fransen J, Jansen TL, Neogi T, Schumacher HR, Taylor WJ. New classification criteria for gout: a framework for progress. Rheumatology. 2013;52(10):1748-53. doi:10.1093/rheumatology/ket154.

16. Wallace KL, Riedel AA, Joseph-Ridge N, Wortmann R. Increasing prevalence of gout and hyperuricemia over 10 years among older adults in a managed care population. J Rheumatol. 2004;31(8):1582-7.

17. Nan H, Qiao Q, Dong Y, Gao W, Tang B, Qian R et al. The prevalence of hyperuricemia in a population of the coastal city of Qingdao, China. J Rheumatol. 2006;33(7):1346-50.

18. Miao Z, Li C, Chen Y, Zhao S, Wang Y, Wang Z et al. Dietary and lifestyle changes associated with high prevalence of hyperuricemia and gout in the Shandong coastal cities of Eastern China. J Rheumatol. 2008;35(9):1859-64.
19. Malik A, Dinnella JE, Kwoh CK, Schumacher HR. Poor validation of medical record ICD-9 diagnoses of gout in a veterans affairs database. J Rheumatol. 2009;36(6):1283-6. doi:10.3899/jrheum.081195.
20. Harrold LR, Saag KG, Yood RA, Mikuls TR, Andrade SE, Fouayzi H et al. Validity of gout diagnoses in administrative data. Arthritis Rheum. 2007;57(1):103-8. doi:10.1002/art.22474.
21. Roddy E, Zhang W, Doherty M. Concordance of the management of chronic gout in a UK primary-care population with the EULAR gout recommendations. Ann Rheum Dis. 2007;66(10):1311-5. doi:10.1136/ard.2007.070755.
22. Roddy E, Mallen CD, Hider SL, Jordan KP. Prescription and comorbidity screening following consultation for acute gout in primary care. Rheumatology. 2010;49(1):105-11. doi:10.1093/rheumatology/kep332.
23. Ludvigsson JF, Andersson E, Ekbom A, Feychting M, Kim JL, Reuterwall C et al. External review and validation of the Swedish national inpatient register. BMC public health. 2011;11:450. doi:10.1186/1471-2458-11-450.
24. Socialstyrelsen. Kodningskvalitet i patientregistret - Ett nytt verktyg för att mäta kvalitet. 2013.
25. Linnersjo A, Hammar N, Gustavsson A, Reuterwall C. Recent time trends in acute myocardial infarction in Stockholm, Sweden. International journal of cardiology. 2000;76(1):17-21.
26. Turesson C, Jacobsson L, Bergstrom U. Extra-articular rheumatoid arthritis: prevalence and mortality. Rheumatology. 1999;38(7):668-74.
27. Harrold LR, Yood RA, Andrade SE, Reed JI, Cernieux J, Straus W et al. Evaluating the predictive value of osteoarthritis diagnoses in an administrative database. Arthritis Rheum. 2000;43(8):1881-5. doi:10.1002/1529-0131(200008)43:8<1881::AID-ANR26>3.0.CO;2-#.
28. Katz JN, Barrett J, Liang MH, Bacon AM, Kaplan H, Kieval RI et al. Sensitivity and positive predictive value of Medicare Part B physician claims for rheumatologic diagnoses and procedures. Arthritis Rheum. 1997;40(9):1594-600. doi:10.1002/1529-0131(199709)40:9<1594::AID-ART8>3.0.CO;2-H.

Tables

Table 1 *Overview of the criteria and cutoffs used in historical algorithms to diagnose gout*

**Criteria**	**Rome 1963**	**New York 1966**	**ARA 1977**	**Mexico 2010**	**Netherlands 2010**
**Cutoff for gout classification**	**≥2 of 4 criteria**	**≥2 of 4 criteria**	**6 of 12 criteria OR presence of MSU in SF**	**4 of 8 criteria OR presence of MSU in SF**	**>8 points***
Serum uric acid, µmol/L,	Male >420 Female >360		>2 SD normal	>2 SD normal	>350 (3.5 points)
Presence of tophi	X	X	X	X	X (13 points)
MSU crystals in SF or tissue	x		(x)		
History of attacks of painful joint swelling with abrupt onset and resolution within 2 weeks	≥2 attacks	≥2 attacks			
A history or observation of podagra		X		X	
Rapid response to colchicine treatment, defined as a major reduction in the objective signs of inflammation within 48 h		X			
More than one attack of acute arthritis			X	X	
Maximum inflammation developed within 1 day			X	X	X (0.5 point)
Oligoarthritis attack			X		
Redness observed over the joints			X	X	X (1 point)
First MTP joint painful or swollen			X		
Unilateral first MTP joint attack			X		
Unilateral tarsal joint attack			X	X	
Asymmetrical swelling within a joint on X-ray			X		
Subcortical cysts without erosions on X-ray			X		
Joint culture negative for organisms during attack			X		
Mono and/or oligoarticular attacks				X	X (2 points)
Male sex					X (2 points)
MTP1 involvement					X (2.5 points)
Hypertension or more than one cardiovascular disease**					X (1.5 points)

*A summed score of 4 or less excludes gout; 8 or more suggests gout; between 4 and 8 suggests the need for SF analysis

**Cardiovascular disease was defined as angina pectoris, myocardial infarction, heart failure, cerebrovascular accident, transient ischemic attack or peripheral vascular disease

Abbreviations: MSU, monosodium urate; MTP, metatarsophalangeal; SF, synovial fluid

Table 2 *Demographic and clinical characteristics of patients in the primary care and rheumatology groups*

Patient characteristics	Primary care group n=262		Rheumatology group n=132	
	Men n=198	Women n=64	Men n=106	Women n=26
Age, years median (range)	63.5 (31–97)	66.5 (43–98)	66.5 (26–91)	71 (34–94)
No. with hypertension (%)	110 (56)	45 (70)	68 (64)	18 (69)
No. with cardiovascular disease (%)*	61 (31)	27 (42)	44 (42)	10 (39)
No. with diabetes (%)*	41 (21)	20 (31)	21 (20)	4 (15)
No. with allopurinol treatment ever (%)*	119 (60)	41 (64)	81 (77)	15 (58)

*Comorbidity was considered to be present if it was mentioned in the clinical record or medication for the comorbidity was prescribed.

Table 3 *Frequencies of patients who had at least one, two, or three gout diagnoses and who met the cutoffs of the different classification criteria*

Classification criteria sets	Primary care group			Rheumatology group		
	n=262	n=84	n=27	n=132	n=83	n=62
	≥1 ICD-10 gout	≥2 ICD-10 gout	≥3 ICD-10 gout	≥1 ICD-10 gout	≥2 ICD-10 gout	≥3 ICD-10 gout
Rome, n	18	16	8	84	61	46
PPV%	7	19	30	64	73	74
95% c.i.	4- 10	11-27	13 - 47	56 - 72	63 - 83	63 - 85
New York, n	9	7	3	80	57	44
PPV%	3	8	11	61	69	71
95% c.i.	1 - 5	2 - 14	0 - 22	53 - 69	59 - 79	60 - 82
ARA, n	54	45	19	90	65	51
PPV %	21	54	70	68	78	82
95% c.i.	16 - 26	43 - 65	53 - 87	60 - 76	69 - 87	72 - 92
Mexico, n	106	68	24	105	73	54
PPV%	40	81	89	80	88	87
95% c.i.	34 - 46	73 - 89	77 - 101	73 - 87	81 - 95	79 - 95
Netherlands,n	110	67	24	94	63	43
PPV%	42	80	89	71	76	69
95% c.i.	36 - 48	71 - 89	77 - 101	63 - 79	67 - 85	57 - 81

Data in the table are the positive predictive values (PPVs) with the corresponding 95% confidence interval (*ci)*

Table 4 *Number of patients displaying gout characteristics*

Gout characteristics	Primary care group (n=262)		Rheumatology group (n=132)	
	Men n=198 (%)	Women n=64 (%)	Men n=106 (%)	Women n=26 (%)
Increased[a] serum uric acid	139 (70/89) [1,b]	41 (64/87) [1,c]	82 (77/80) [1,d]	15 (58/65) [1, e]
Analysis of MSU crystals in SF (%)	6 (3)	0	34 (32)	1 (4)
Presence of tophi (%)	3 (2)	3 (5)	27 (23)	4 (15)

[a] Male >420 µmol/L, female >360 µmol/L
[1] Percentage defined as the proportion with abnormally high values out of: 1) all patients reviewed and 2) those with an available test results
[b] 41 missing values
[c] 17 missing values
[d] 3 missing values
[e] 4 missing values

Table 5 *The five most commonly reported symptoms of gout in the medical records*

Symptom	Total (n=394)	Primary care group, n=262 (%)		Rheumatology group, n=132 (%)	
		Men n=198	Women n=64	Men n=106	Women n=26
Monoarthritis	238 (60)	130 (66)	36 (56)	59 (56)	13 (50)
First MTP arthritis	220 (56)	115 (58)	29 (45)	64 (60)	12 (46)
Redness over joints	193 (49)	98 (50)	31 (48)	52 (51)	12 (46)
≥2 or more attacks of arthritis	185 (47)	62 (31)	15 (23)	91 (86)	17 (65)
Unilateral first MTP arthritis	178 (45)	98 (50)	24 (38)	46 (43)	10 (39)

Supplementary Table 1 *Frequencies of patients who had one, two, or more main gout diagnoses and who met the cutoffs of the different classification criteria.*

Classification criteria sets	Primary care group			Rheumatology group		
	n=119	n=84	χ^2-test 1 vs ≥2 p-value	n=49	n=83	χ^2-test 1 vs ≥2 p-value
	1 ICD-10 gout	≥2 ICD-10 gout		1 ICD-10 gout	≥2 ICD-10 gout	
Rome, n	2	16	<0.0001	23	61	0.004
PPV,%	2	19		47	73	
95% c i	-1 - 5	11 - 27		33 - 61	63 - 83	
Age, median	77	71		73	64	
New York, n	2	7	0.03*	23	57	0.02
PPV,%	2	8		47	69	
95% c i	-1 - 5	2 - 14		33 - 61	59 - 79	
Age, median	78	86		67	64	
ARA, n	9	45	<0.0001	25	65	0.002
PPV,%	8	54		51	77	
95% c i	3 - 13	43 - 65		37 - 65	68 - 86	
Age, median	76	73		70	64	
Mexico, n	38	68	<0.0001	32	73	0.004
PPV,%	32	81		65	88	
95% c i	24 - 40	73 - 89		52 - 78	81 - 95	
Age, median	67	68		69	64	
Netherlands,n	42	67	<0.0001	31	63	0.2
PPV,%	35	80		63	76	
95% c i	26 - 44	71 - 89		49 - 67	67 - 85	
Age, median	70	71		71	64	

PPV with 95% confidence interval (ci) and median age in years

** Fisher's exact test*
